# Tailoring One-Pass Asymmetric Rolling of Extra Low Carbon Steel for Shear Texture and Recrystallization

**DOI:** 10.3390/ma12121935

**Published:** 2019-06-15

**Authors:** Satyaveer Singh Dhinwal, Laszlo S. Toth, Rimma Lapovok, Peter Damian Hodgson

**Affiliations:** 1 Laboratory of Excellence on Design of Alloy Metals for Low-Mass Structure (Labex-DAMAS), Université de Lorraine, 57070 Metz, France; 2 Université de Lorraine, CNRS, Arts et Métiers ParisTech, LEM3, F-57000 Metz, France; 3 Institute for Frontier Materials (IFM), Deakin University, Geelong, VIC 3216, Australia; r.lapovok@deakin.edu.au (R.L.); peter.hodgson@deakin.edu.au (P.D.H.)

**Keywords:** asymmetric rolling, texture, thickness reduction per pass, shear coefficient, recrystallization, steel

## Abstract

Systematic single pass rolling experiments were carried out at room temperature on extra low carbon steel by varying the roll diameter ratio between 1:1 to 1:2 and thickness reduction per pass in the range of 20–75%. The aim of this study was to define the conditions under which the rolling texture can transit into a shear texture. The consequences for grain fragmentation, tensile strength, recrystallization texture, and grain growth kinetics were also studied. It was found that in a certain range of thickness reduction per pass and asymmetric ratio, an effective rotation towards the shear texture takes place in conventional rolling. The value of the shear coefficient factor (shear strain rate/rolling strain rate) in asymmetric rolling depends on the selection of thickness reduction per pass. The measured value of shear coefficient was found to be independent of the number of passes used in asymmetric rolling. The consequence of arising shear textures is an acceleration of grain fragmentation. After rapid heat treatment, both tensile strength and recrystallization kinetics of asymmetric rolled sheets showed merits over the conventional rolling. Only the evolved Goss orientation from asymmetric conditions of deformation shows higher stability than any other preferred shear texture components after complete recrystallization.

## 1. Introduction

In recent years, rolling with imposed asymmetry has gathered interest to introduce a through thickness shear texture effect in a rolled sheet [1,2,3,4,5,6,7,8,9,10,11]. It has been also considered that imposing asymmetric conditions just by varying the rolling parameters in conventional rolling is the most efficient way to introduce shear into the sheet [12,13,14]. Several studies report that the added shear strain in rolling can lead to increased grain refinement, tensile strength improvement, and tilt/rotation of the rolling texture, so one can tailor the deformation texture anisotropy [1,4,6,15,16,17]. Based on the type of applied asymmetry, different nomenclatures have been given to asymmetric rolling processes [4,5,9,10,15,16]. There are three ways to introduce asymmetry: difference in roll diameter, in angular speed, or in friction. However, the pre-notion was that the effect of these different ways of imposed asymmetry in rolling remains mostly similar on deformed texture and microstructure, except the tribological type of asymmetry, which is reported to be much less effective than the others [18].

It has been predicted from numerical methods that the shear effect generated from the applied asymmetric conditions can tilt/rotate the conventional rolling texture up to the ideal shear texture, or close to it [2,3,11,19]. It was reported in these works that the asymmetric ratios, such as 1:1.5 or above, can tilt/rotate texture significantly when the applied thickness reduction per pass (TRPP) is small or medium (5–40%) in multi-pass rolling. It was also found in experiments on multi-pass asymmetric rolling that the texture in the mid thickness area of the sheet remains nearly the same as for symmetric rolling [2,3,11,19]. However, in a very recent publication, current authors have shown the possibility of forming a through thickness shear texture in multi-pass rolling when all parameters are high—thickness reduction per pass, diameter ratio, and total thickness reduction [20]. It was also found in that work that by simple rigid body rotation the conventional rolling texture turns into shear texture. However, it is yet to investigate the dependence of texture rotation on the applied number of passes and the thickness reduction per pass.

A range of shear coefficient (defined as the ratio of shear strain rate/rolling strain rate) values has been considered in various investigations to predict the texture rotation in asymmetric rolling [2,3,6,11,19,21]. However, so far, this parameter has not been measured experimentally in order to correlate it with the texture rotations in asymmetric rolling.

Huang et al. [22] carried out single pass asymmetric rolling on magnesium alloys. They found little rotation of the texture in spite of the large reduction of passes, up to 63%. This, however, is understandable, because of their initial texture, which was a strong basal texture. Namely, such a texture is highly stable in simple shear [23], so it is not expected to rotate under the shear component of the asymmetric rolling process. Several other investigations were also carried out with high ratio differential speed rolling combined with high TRPPs in single pass rolling [16,24,25]. However, these studies were focused on the extreme conditions of asymmetric rolling rather than on the formation of shear textures.

The present study aims to reveal texture evolutions as a function of thickness reduction per pass. Single pass asymmetric rolling was carried out on extra low carbon steel. The effect of texture rotation was also examined in terms of microstructure evolution. We selected a ferritic steel because there are only a few investigations about its behavior in asymmetric conditions. Shear texture plays a very important role in several applications. For example, with added silicon content in ferritic steel, a through thickness shear texture in rolled sheet is the prime requirement to minimize eddy current loss in transformers.

## 2. Materials and Methods

Specimens with the dimensions of 30 mm width × 60 mm length and 5 mm in thickness were machined from a billet which was earlier heat treated at 1000 °C for an hour. The front end of the specimens was wedged to ease achieving a high thickness reduction per pass (TRPP). In some specimens, vertical pins were inserted, made of the same material. This step was taken in order to measure the value of the shear coefficient from the inclination of the pins, as reported in other investigations [7], and associate it with the texture evolution.

**Figure 1:**
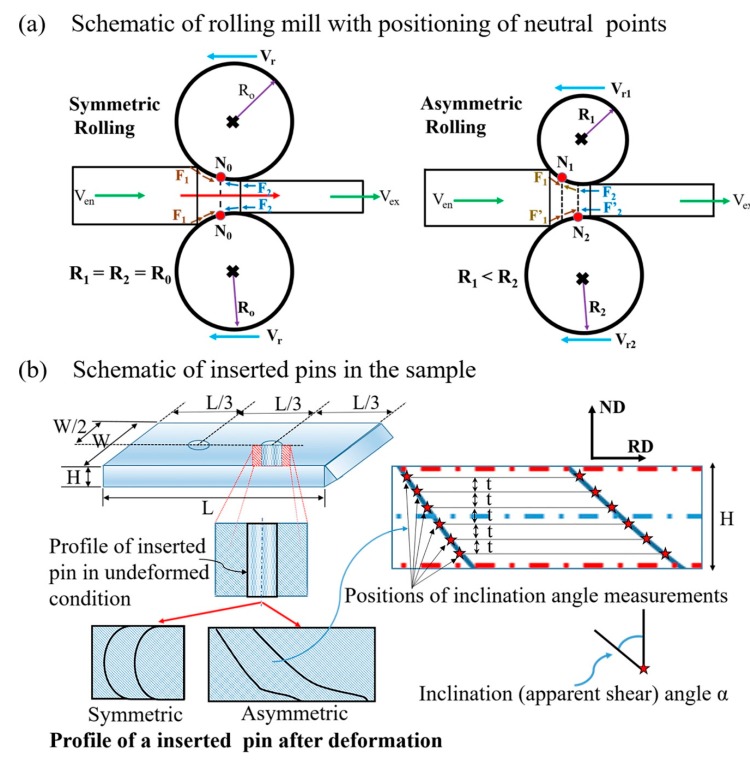
Schematic of the experimental setup, representing positons of (**a**) neutral points and (**b**) inserted pins in a sample.

In addition to the insertion of pins, a vertical grid was also made using indentation marks, forming a square mesh of 0.75 mm × 0.75 mm on the TD (Transverse Direction) plane at the edge of the specimen. In this way, the results obtained from both inserted pins and grids could be compared. For multiple passes, additional new vertical inserts were placed into the sheets before each pass. A schematic of the experimental setup is presented in Figure 1, showing the positions of the neutral points and the reference system.

Rolling was carried out at room temperature with special attention paid to the rolls, maintaining them in a smooth and dry state for every pass. The sheets were rolled in monotonic mode without strain path change. The asymmetric conditions were imposed by differences in roll diameter ratio for the values of 1:1.3, 1:1.6, 1:2. The TRPP was varied between 20% and 75%. A single motor driven Carl Wezel rolling mill was equipped with a special roll-set for the experiments. It had four sets of diameter ratios, from 1:1 (200 mm/200 mm) to 1:2 (133 mm/267 mm), and their revolutions per minute was 32. The results were compared with the symmetric (1:1) case, keeping the other parameters the same as the asymmetric case. Table 1 shows the carried out rolling schedule. Note that due to the spring back effect of the rolling mill, the final thickness reduction of a sheet at each TRPP was always less than the scheduled TRPP; the differences went up to 7%.

For X-ray diffraction and electron back scattered diffraction (EBSD), samples from the rolled sheet were first mechanically polished with 600, 1200, and 1800 SiC grit papers, before further polishing steps. Thereafter, the samples were polished with 6 µm, 3 µm, and 1 µm diamond suspension and final polishing was performed with colloidal silica suspension (OP-S solution).

The bulk texture of the rolled specimens were measured by X-ray diffraction on the ND (Normal Direction) plane at mid thickness, as well as about 200 µm below the top and bottom surfaces of the sheet. The Labosoft™ software [26] was used for texture data analysis without applying any sample symmetry. A total of 10° deviation from the ideal position was considered for the volume fraction quantification of texture components. The deformed microstructure was examined in the mid thickness area of the TD surface by a scanning electron microscope (SEM) equipped with an electron back scattered diffraction (EBSD) detector. The SEM for EBSD was operated with 15 kV voltage. The step size for scanning was chosen between 0.35 and 0.2 µm based on the features of interest in the microstructure. The noise reduction in the scanned EBSD maps was carried out by removing the first wild spike, followed by the standard procedure of assigning the minimum six neighboring pixel’s orientation to the non-indexed pixels with repetitive iterations. The microstructural representation and quantification of data collected from the EBSD-scanned area were analyzed using the Channel 5 software provided by Oxford Instruments. The ATEX software [27] was also used for data post-processing. The reference system for the Euler angles of the crystal orientations were defined as: RD = x, TD = y, ND = z on Inverse Pole Figure (IPF) maps. The grains were defined on the basis of a minimum disorientation angle of 5° and consisted at least of three or more pixels.

For recrystallization kinetics study, isothermal annealing at 660 ± 10° temperature was used. Material strength before and after recrystallization was measured by tensile testing at a 0.006 mm/second strain rate with specimen dimensions down sized from the ASTM E8 standard size. The tensile specimens were grinded and polished sufficiently enough from both sides of top and bottom surfaces so that deformation effects arising due to change in roll diameter ratios could be examined more precisely in the mid thickness region of the rolled sheet.

## 3. Results

**Figure 2:**
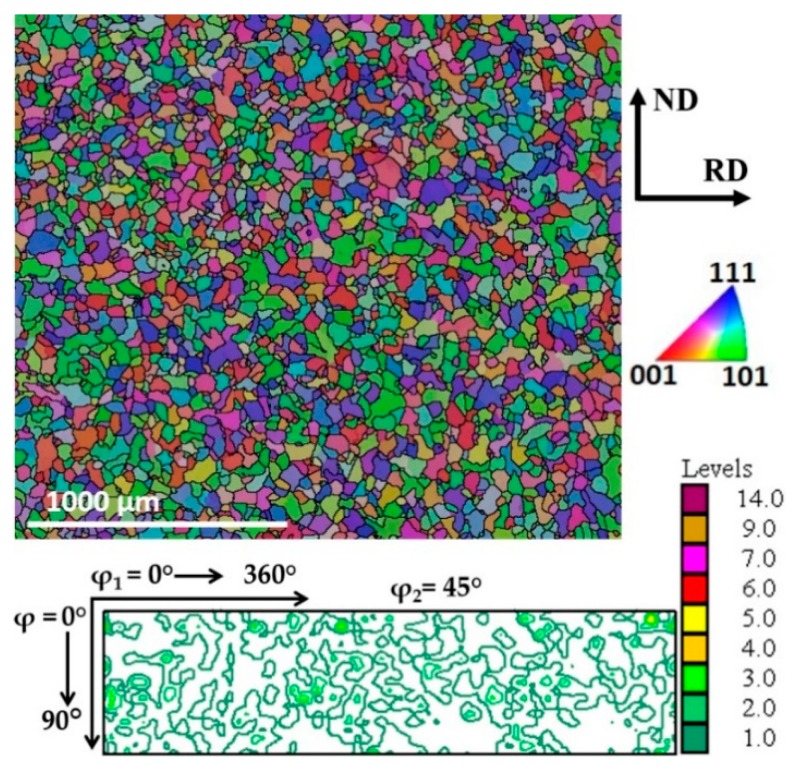
The initial microstructure (EBSD IPF map) and texture (the φ_2_ = 45° ODF section) of extra low carbon steel after heat treatment.

**Figure 3:**
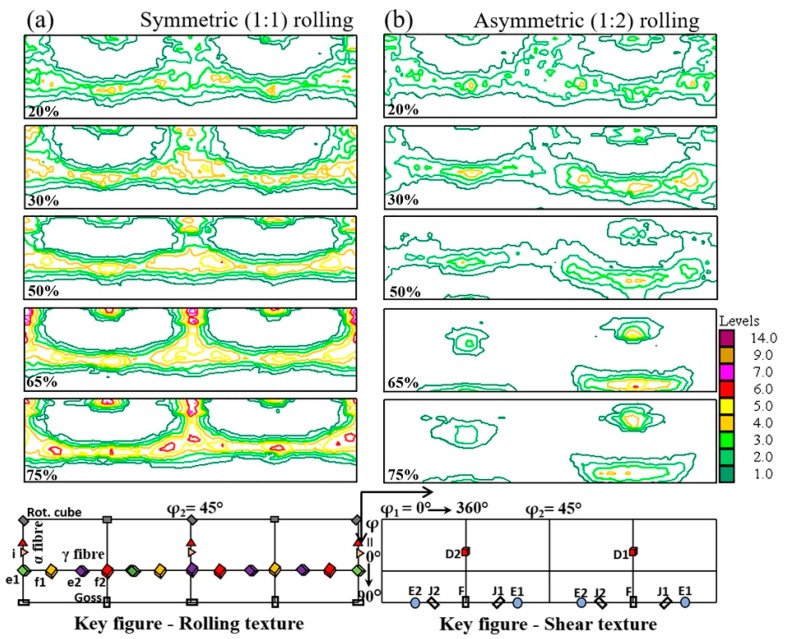
φ_2_ = 45° sections of ODFs in mid thickness area for (**a**) symmetric (1:1) and (**b**) asymmetric (1:2) single pass rolling with a thickness reduction per pass (TRPP) in the range of 20–75%.

Figure 2 shows the microstructure and texture of the initial material. The microstructure after the prior heat treatment to rolling consisted of equiaxed ferrite grains with an average size of about 36 µm, and a texture that was practically random. Figure 3 represents the measured bulk texture in the φ_2_ = 45° sections of the orientation distribution function (ODF) at the mid thickness area of a sheet for rolling with diameter ratios of 1:1 and 1:2, with TRPP increasing from 20% to 75%, in single passes. It is apparent that the texture development depends both the TRPP and the roll diameter ratio. For symmetric rolling, the texture intensity steadily increased and the α and γ fibres became more defined. Whereas at the roll ratio of 1:2, these two fibres disappeared completely, while the texture intensity did not change significantly. At the same time, the texture components inclined progressively towards the shear texture components.

**Figure 4:**
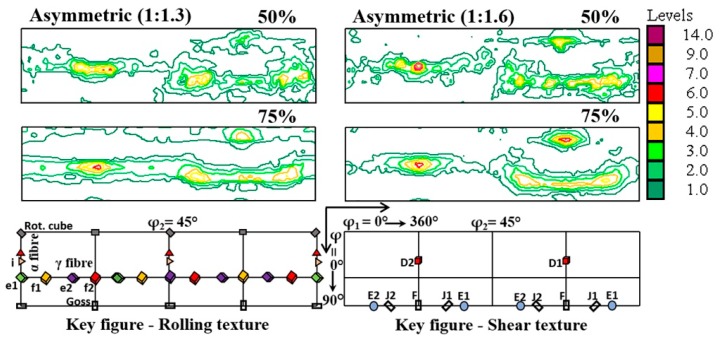
φ_2_ = 45° sections of ODFs in the mid thickness area for asymmetric ratios of 1:1.3 and 1:1.6 with a TRPP of 50% and 75%, in single pass.

Figure 4 shows the resultant texture at the intermediate ratios of 1:1.3 and 1:1.6 for a thickness reduction per pass of 50% and 75%, in single pass. The loss of texture symmetry with respect to symmetric rolling is visible by the displacement of the second part of the γ fibre (extending from φ_1_ = 180° to 360°) for both roll diameter ratios. At the same time, the shifted fibre part becomes more defined by increasing the TRPP value. The displacement of this rotated fibre was higher for the 1:1.6 asymmetry ratio, nevertheless, remaining inferior to what was observed at the ratio 1:2 in Figure 3.

**Figure 5:**
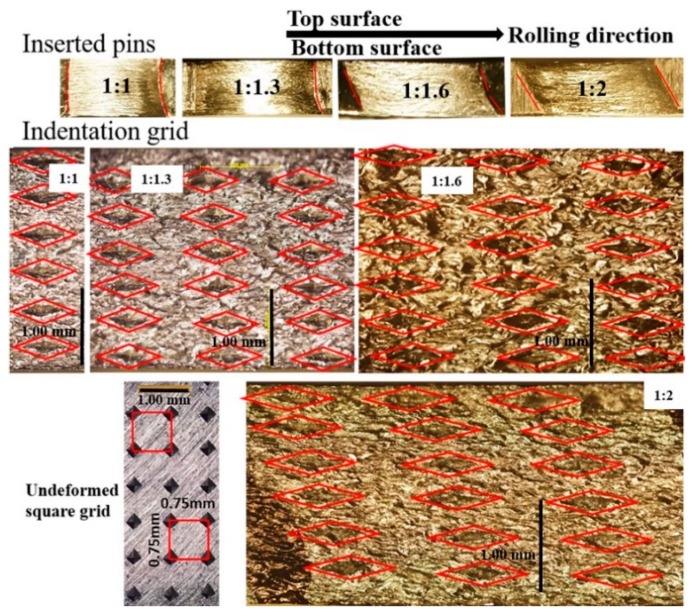
Changes in the geometry of the inserted pins and indentation marks with increasing roll diameter ratio at the same TRPP value (50%).

**Figure 6:**
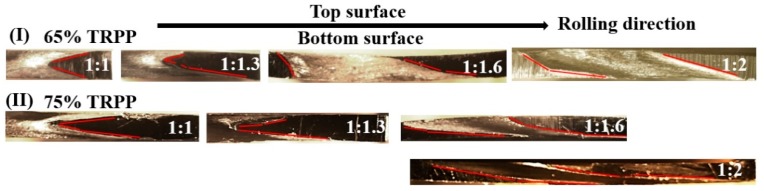
Change in inclination profile of pins at ratios 1:1–1:2 with TRPPs of 65% and 75% for the whole sheet thickness.

The effect of asymmetry was examined at a constant (50%) TRPP, with the help of inserted pins and indentation marks; the metallographic images are shown in Figure 5. Their shape change reflects the imposed strain field. The shear component changed the orientation of a vertical line. The red lines in Figure 5 and Figure 6 indicate the shapes of the markers. In symmetric rolling, the shear component changes the sign between the top and bottom half of the sample in a symmetric manner. By introducing dissymmetry in the roll diameters, the bottom shear zone extends upward. From the ratio 1.6, it occupies the whole sample.

**Figure 7:**
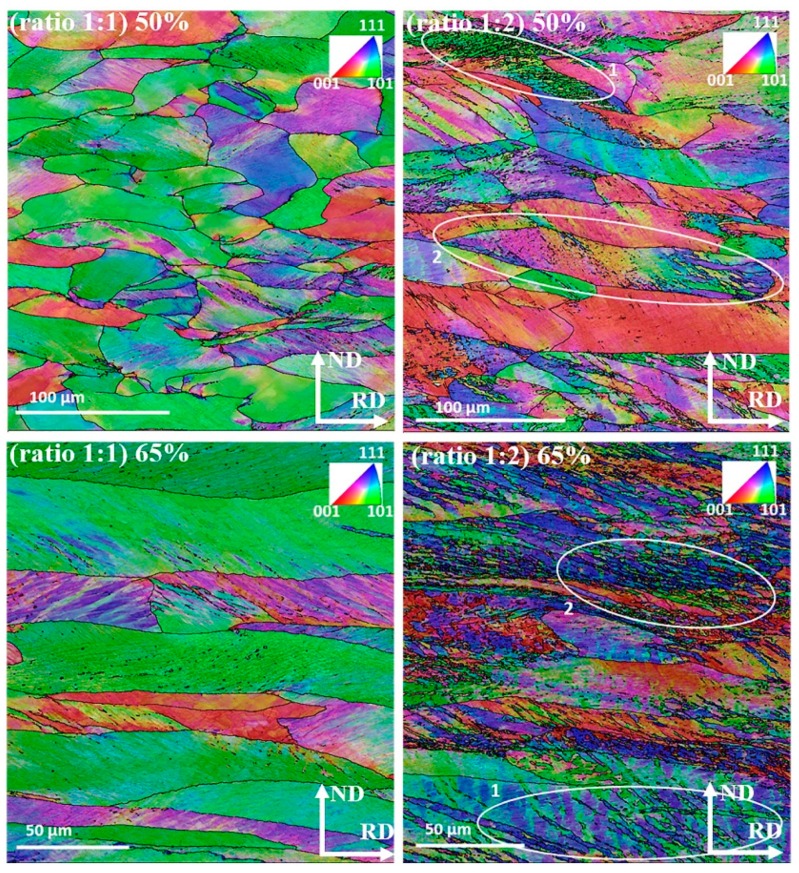
IPF maps taken on the TD surface (by projecting the RD axis), in the mid thickness area, for asymmetry ratios of 1:1 and 1:2 with TRPPs of 50% and 65%.

**Figure 8:**
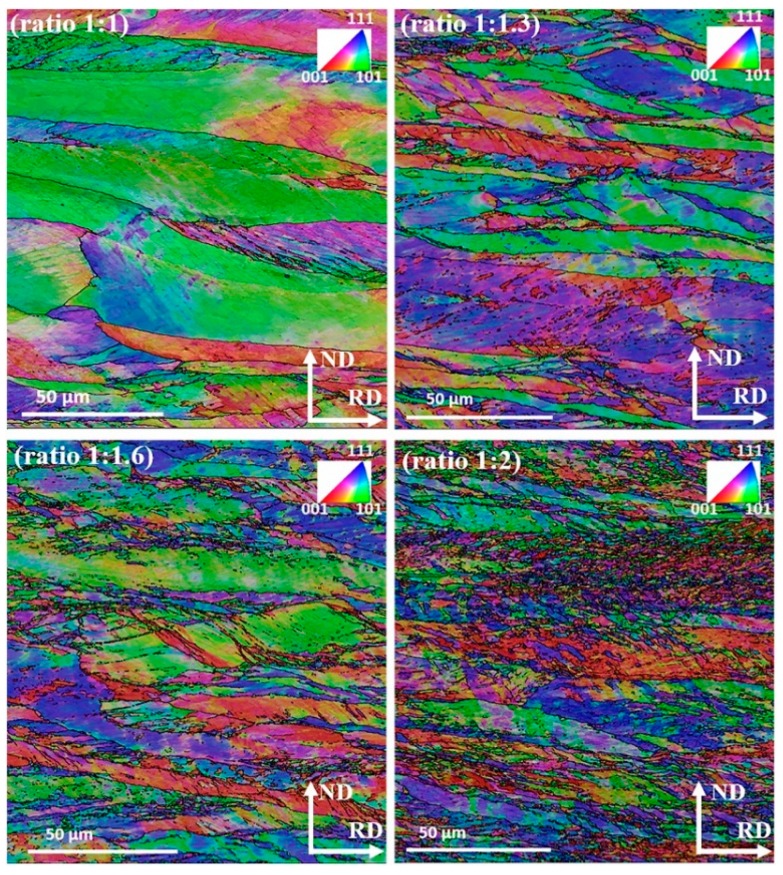
IPF maps taken on the TD surface (by projecting the RD axis), in the mid thickness area of a sheet at TRPP of 75% with an increase in the roll diameter ratio from 1:1 to 1:2.

The signature of the imposed asymmetric condition is quite evident in the measured microstructures taken from the mid thickness area on the TD plane, see Figure 7 and Figure 8. These figures show inverse pole figure (IPF) maps of the RD axis, where the grain boundaries with disorientations larger than 15° were superimposed. It is noticeable that with increase in TRPP, the microstructure becomes more advanced at the asymmetric ratio of 1:2 than at any other applied ratios.

**Figure 9:**
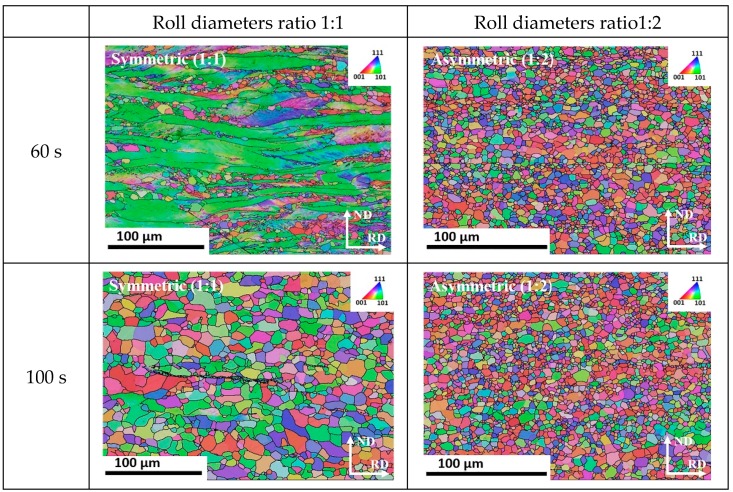
Recrystallization IPF maps (TD surface) in the mid thickness area of a sheet at 660 ± 10 °C for 75% thickness reduction per per pass.

**Figure 10:**
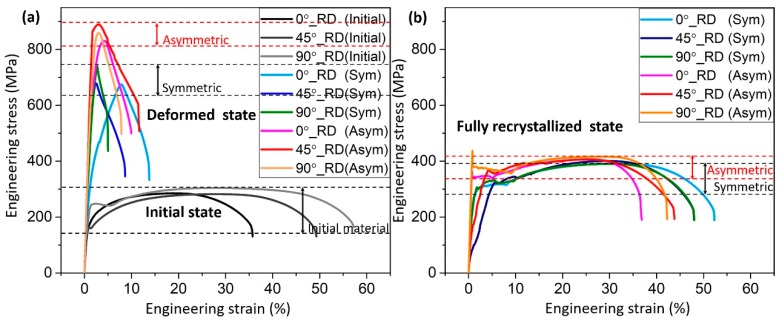
Engineering stress–strain curves in tensile testing after deformation by 75% thickness reduction in a pass (**a**) and recrystallization (**b**) for 100 s at 660 ± 10 °C.

Figure 9 represents the microstructure obtained in isothermal recrystallization conditions for asymmetry ratios of 1:1 and 1:2, for 60 s and 100 s annealing times. One can observe faster complete recrystallization at the asymmetry ratio 1:2 for 60 s. Figure 10 shows the mechanical behaviour in tensile testing before and after recrystallization for samples rolled to 75% thickness reduction in one pass.

## 4. Discussion

This study shows that the selection of applied thickness reduction per pass for a given asymmetry ratio strongly affects both texture and microstructure development. In the following, the discussion is sub-sectioned in three parts to examine the effect of asymmetric conditions on:

1. shear strain distribution and texture rotation,
2. deformed microstructure,
3. recrystallization kinetics, texture and tensile strength.

### 4.1. Shear Strain Distribution and Texture Rotation

The shear coefficient *p* was defined in Reference [7] as the ratio of the shear strain rate $\left(\dot{\gamma }\right)$ and the rolling strain rate $\left(\dot{\varepsilon }\right):$

(1) $$\dot{\gamma }=-p\dot{\varepsilon }.$$

*p* is constant if the velocity gradient remains constant during rolling. In this case, after one pass, or after any given number of passes for an initially vertical pin, the *p* value is determined by the initial ($h_{i}$) and final ($h_{f}$) thicknesses of the sheet by:

(2) $$p=\left(\frac{h_{f}}{h_{i}-h_{f}}\right)\tan\left(\alpha \right),$$

where α is the inclination angle of the inserted pin after each rolling pass with respect to its initial vertical position, $h_{i}$ is the sheet thickness prior to a given pass, and $h_{f}$ is the thickness after the pass. From Equations (1) and (2), we obtained the shear strain value:

(3) $$\gamma =\left(\frac{h_{f}}{h_{i}-h_{f}}\right)\tan\left(\alpha \right)\ln\left(\frac{h_{f}}{h_{i}}\right).$$

**Figure 11:**
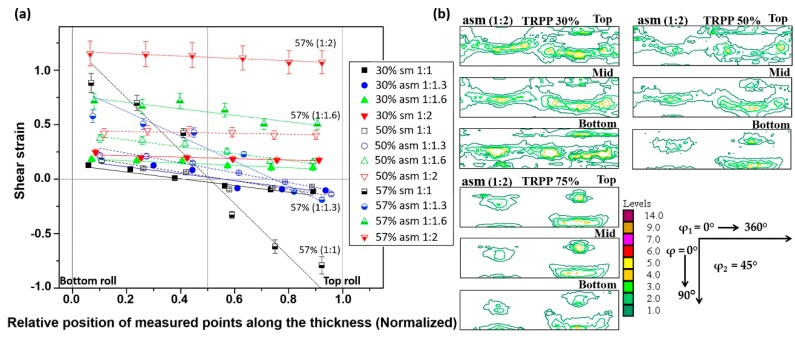
(**a**) Shear strain distribution at 30–57% thickness reduction per pass with roll diameter ratios of 1:1–1:2. (**b**) ODF sections for the asymmetry ratio 1:2 with thickness reduction per pass of 30–75%.

This relationship shows that the shear strain is function of both the applied sheet thickness reduction and the inclination angle of the initially vertical pin in a pass. Note that this equation can be employed locally—namely, α is not necessarily constant through the thickness (see Figure 5; Figure 6). The results of such analysis are presented in Figure 11a. It is clear from this Figure 11a that the shear strain distribution depends both on the roll diameter ratio and on the TRPP. The shear strain is practically uniform through the thickness when the roll diameter ratio is 1:2 compared to other applied ratios.

Shear values and shear coefficients were reported for asymmetric rolling in several papers [3,6,28,29,30,31]. The shear strain distributions within the thickness were examined experimentally in Reference [29] with the help of indentation marks, and by simulations in [29,30,31,32,33,34,35]. The main finding of Roumina et al. [29] was that higher TRPPs in asymmetric rolling lead to more uniform shear distribution in multi-passes for the same total thickness reduction. Our results in Figure 11a also show that for a given TRPP, both the magnitude of the shear strain and its uniformity through the thickness are higher by increasing the asymmetry ratio.

It has been shown in References [29,34] that the neutral point (see in Figure 1) is shifted opposite to RD on the top roll and towards RD at the bottom roll in asymmetric rolling. In this way, a cross-shear zone is formed, in which both shears produced at the top and bottom rolls are shearing the metal in the same way. This is not the case for symmetric rolling leading to two shear zones with opposite shears (see Figure 5 and Figure 6). This means that asymmetric rolling is promoting deformation homogeneity within the sheet. Our results clearly show that the obtained shear component depends on both TRPP and on the asymmetry ratio. These two parameters are playing decisive roles in increasing the size and the effectiveness of the cross-shear zone. However, to reach high shear it is vital to impose a high asymmetry ratio combined with high TRPP. We emphasize that a high TRPP with low asymmetry cannot produce the same result.

**Figure 12:**
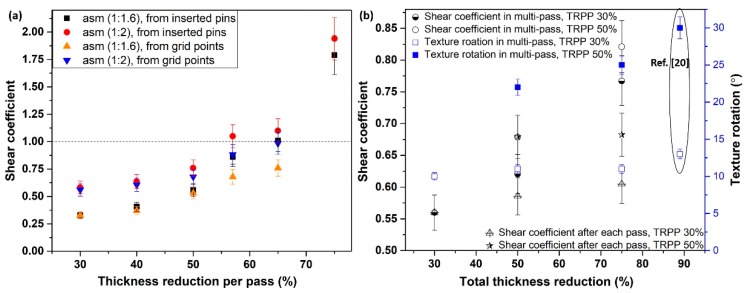
(**a**) Shear coefficient measurement in the mid thickness area of a sheet for single pass rolling with TRPP of 30–75% and asymmetry ratios 1:1.6–1:2, and (**b**) for a total thickness reduction of 75% in multi-passes with TRPP of 30% and 50% at the asymmetry ratio of 1:2.

The texture rotation due to shear is strongly affected by the combination of the TRPP and the asymmetry ratios. As can be seen in Figure 11b, significant texture rotation is only obtained when both TRPP and asymmetry ratio are high. Under such conditions, along with the uniform shear strain distribution, a uniform rotation of the texture is obtained, leading to a homogeneous shear texture across the thickness in asymmetric rolling. It follows from the texture results (Figure 3) and from the measured shear coefficient trends (Figure 12a), that after reaching a certain range of parameters in the asymmetric rolling conditions, the amplitude of the shear strain can become comparable to the rolling strain in the mid thickness area of the sheet (Figure 3 and Figure 12a).

Shear coefficients were also determined for multiple passes, see the results in Figure 12b. There were two possibilities in multiple passes to obtain shear coefficient values. One way was to use the initial inserts, and the other was to use new inserts, which were placed additionally before each new pass. These two ways should have led to the same result using the proper initial and final thickness values. However, as can be seen in Figure 12b, the shear coefficients obtained from the new inserts were systematically smaller than those derived from the initial pins. Moreover, they were nearly constant in each pass. This discrepancy actually means that Equation (1) is not fulfilled in asymmetric rolling, so Equations (2) and (3) are also not valid. The obtained differences can be only explained by assuming that in a given pass the shear is not happening at the same time as the reduction in thickness (the rolling strain). It is quite probable that the deformation process begins with the reduction of thickness without any significant shear, followed by a new stage, where the thickness is not decreasing significantly, while mostly shear deformation is produced.

Figure 12b displays also the rotation of the texture due to shear. The rotation value was identified by the rotation of the D1 shear texture component, which originates from the rotated cube of the rolling texture (see more on this in Reference [20]). It can be seen that the rotation is limited to 11° when the TRPP is 30%, while it goes up to 30° when the TRPP is 50%. The value of 30° is the limiting value because at this value the rolling texture components turn into the shear ideal texture components (see Reference [20]). Larger rotations are not possible because the ideal positions are reached at 30°.

In Figure 12a, one can notice that the approximation of the shear coefficient from inserted pins and marked grids varied with an increase in TRPP for a given asymmetric ratio. The values obtained from inserted pins were slightly higher than those obtained from the marked grid points. These differences were mostly due to either the unavoidable overestimation of the inclination angles from inserted pins or underestimation of angles using the grids. However, the trend of both results is consistent, considering the error bars (about 10%). Starting from the shear coefficient value of 1.0, Toth et al. [19] have shown by simulation that significant rotation takes place in symmetric rolling texture together with limited stability along both the α and γ fibres, which was the case when the asymmetry ratio was 1:2 and TRPP was about 50–57% in the present experiments.

**Figure 13:**
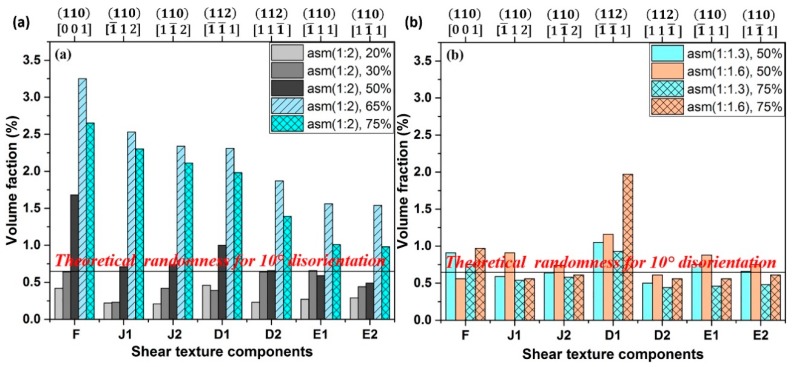
Volume fractions of the prominent shear texture components at (**a**) asymmetry ratio of 1:2 with TRPP of 20–75% and (**b**) at ratios of 1:1.3 and 1:1.6 with TRPP of 50% and 75%.

To obtain shear textures by both the roll diameters, high asymmetry and the TRPP can be evidenced also in the present measurements by studying the volume fractions of the prominent shear texture components. Figure 13 demonstrates that there is a gradual increase in shear texture components at the expense of the rolling components as the TRPP is increased. A noticeable rise in volume fractions of shear texture components above the theoretical randomness occurs only when TRPP is at least 50% together with the asymmetry ratio being larger than 1:1.6.

By combining all the results presented above obtained from the inclination angles of inserted pins and grid marks, the rotation of the texture, and the variation of the shear texture components, it can be concluded that an asymmetry ratio larger than 1:1.6 with TRPP of at least 50% are the criteria for the rolling texture to switch into shear texture.

### 4.2. Deformed Microstructure

The deformed microstructures displayed in Figure 7 and Figure 8 have a common feature concerning the shear bands that appear at about 35° with respect to RD, as a usual microstructure feature in rolling. These bands are significantly accentuated for the asymmetric rolling conditions, in almost all grains. They represent periodic orientation variations between adjacent bands. For the case of 1:2 asymmetry and 65% TRPP, the transformation of the bands into fragmented grain structure is evident. Under the condition of asymmetric deformation, orientations that should remain stable in the symmetric case become unstable, so keep rotating with strain [36,37,38,39,40,41]. Such a scenario can lead to increased grain fragmentation by orientation splitting, deformation bands, and macro shear bands, as compared to the plane strain deformation condition alone. Previous studies have shown that the different types of bands and orientation splitting are appearing as a consequence of the complex imposed deformation state [42,43,44,45]. The increased grain fragmentation for the asymmetric case can be attributed to the strong shear component. Indeed, it has been shown in Reference [46] that grain fragmentation is much more efficient in simple shear compared to pure shear, i.e., symmetric rolling. Thus, the enhanced grain refinement with the imposed severity of asymmetric deformation is due to the increased lattice rotation, an effect well known in simple shear [46].

**Figure 14:**
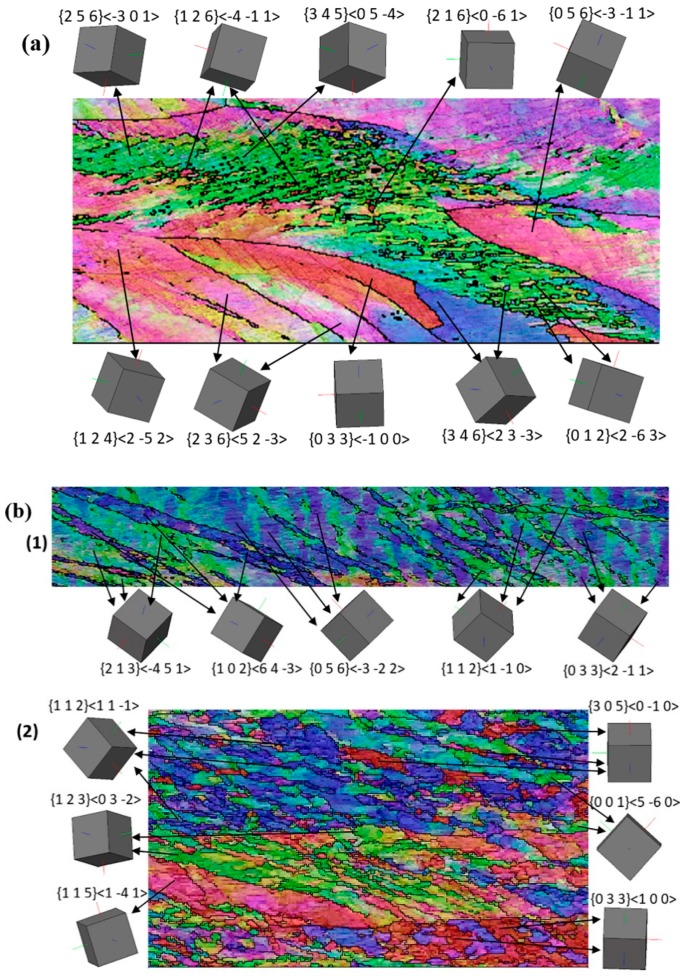
Tendency of local orientations from Figure 7 at an asymmetry ratio of 1:2, (**a**) elliptical marked area 1 at TRPP of 50%, and (**b**) areas 1 and 2 at TRPP of 65%.

Furthermore, examination of what type of orientations can form within these instabilities can be noticed in Figure 14, where local crystal orientations were mapped on selected sites; see in Figure 7 the marked white ellipses. Detailed orientation analysis shows that most of the fragmented sites possess either shear texture orientations or orientations that are different from the parent grain. The formation of such orientations and their gradients by instabilities implies that the asymmetric conditions can promote the development of shear texture, and locally, a randomization of the texture.

### 4.3. Recrystallization Kinetics, Texture, and Tensile Strength

The consequence of imposed asymmetry conditions during deformation also reflects on post processing. The current investigation takes an account of recrystallization behavior in terms of changes in kinetics, texture, and tensile strength.

A perturbed microstructure due to different types of deformation bands formation and their intersections causes heterogeneous distribution of dislocation density and grain boundary junctions in fragmented parts of original grains [45,47,48,49,50,51]. Thereby, an inherent difference lies in accumulated stored energy at different locations in a deformed microstructure or in the distribution of preferential sites for the recrystallization. The deformed microstructure becomes more heterogeneous, with an increase in imposed asymmetric conditions, than the symmetric case in similar conditions of rolling. The newly formed grains grow into a deformed matrix by migration of high angle grain boundaries due to the stored energy difference between them [48,52,53,54]. In the case of asymmetric rolling, there is a higher possibility for these newly formed grains to be surrounded by an increased network of high angle grain boundaries than in symmetric rolling. Because of this, the kinetics of recrystallization are faster in the asymmetric case than in the symmetric one. However, with an increase in time, the coarsening of strain-free new grains can be limited later by their impingement with each other.

**Figure 15:**
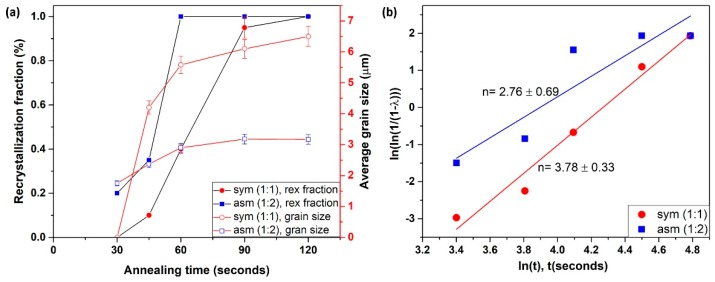
Recrystallization kinetics for symmetric and asymmetric rolling. (**a**) Fraction of recrystallization and average grain size, (**b**) JMAK (Johnson–Mehl–Avrami–Kolmogorov) exponent.

In the case of symmetric rolling, there are still elongated bands of original initial grains; see the green colored bands of α fiber grains in Figure 7 and Figure 8, which retain low angle disorientation ≤7° despite the applied severe deformation. Such grain bands are slower to form new grains than other bands which have already formed strain free grains (example, γ fiber grains) [55]. Interestingly, these low angle disorientation regions of α fiber grains can also be consumed by the growth of existing strain-free grains [56]. Such invasion can lead to a growth of strain-free grains, due to the high mobility of their high angle boundaries [56,57]. Thereby, the occurrence of having a complete recrystallized microstructure with smaller grain size in asymmetric ratio is more probable than in the symmetric case for the same isothermal annealing conditions (Figure 15a). Analysis of the JMAK (Johnson–Mehl–Avrami–Kolmogorov) exponent presented in Figure 15b shows a smaller JMAK value for the asymmetric case than for symmetric rolling. This observation further confirms inhomogeneity both in microstructure and in stored energy distribution, which is increasing the number of nucleation sites for recrystallization [53]. Based on the reduced JMAK exponent and more heterogeneous microstructure, it can be also inferred that the apparent activation energy to initiate recrystallization has also been reduced for the asymmetric case compared to the symmetric one. There is evidence where it was noticed that the apparent activation energy was reduced for a more heterogeneous microstructure [58,59].

**Figure 16:**
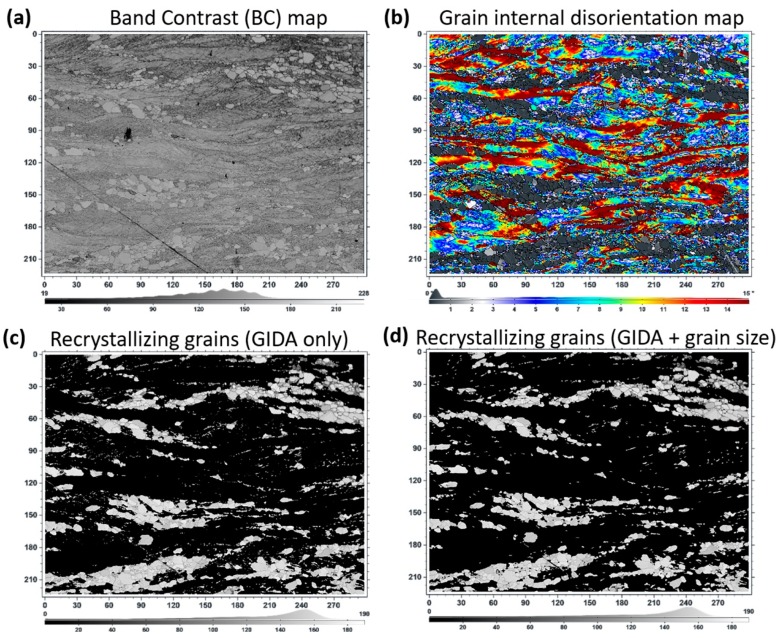
Band contrast map (**a**) and Grain internal disorientation average map (**b**); Partitioning of partially recrystallized microstructure based on the value of the grain internal disorientation average (GIDA) alone, (**c**) as well as with the addition of grain size filter (**d**).

Parameters, such as image quality, band contrast, misorientation average deviation, and grain size, were usually employed to identify the recrystallized grains in the deformed microstructure. However, recently, emphasis has been given more to grain internal disorientation-based partitioning for the recrystallizing grains [52,60,61,62,63,64]. This is due the fact that recrystallizing grains are more dislocation free than the others. Therefore, it is a pragmatic approach to choose a criterion based on the value of the internal disorientation angle below which grains are considered to be recrystallized. Further partitioning based on grain size can also be added for filtering the grain sizes of interest. Figure 16 shows the fraction of recrystallizing grains in the deformed matrix for symmetric rolling as an example of partitioning, based first only on the grain internal disorientation average (GIDA) as well as combined with the grain size filter. It is clear from Figure 16 that the recrystallizing grains go from having a value of zero to a small value of grain internal disorientation average (GIDA) compared to the grains, which are still in the deformed and recovered states. In this case of partially recrystallized sample obtained by symmetric rolling (Figure 16b), the maximum angle of 2.8° was selected to identify the recrystallized grains.

**Figure 17:**
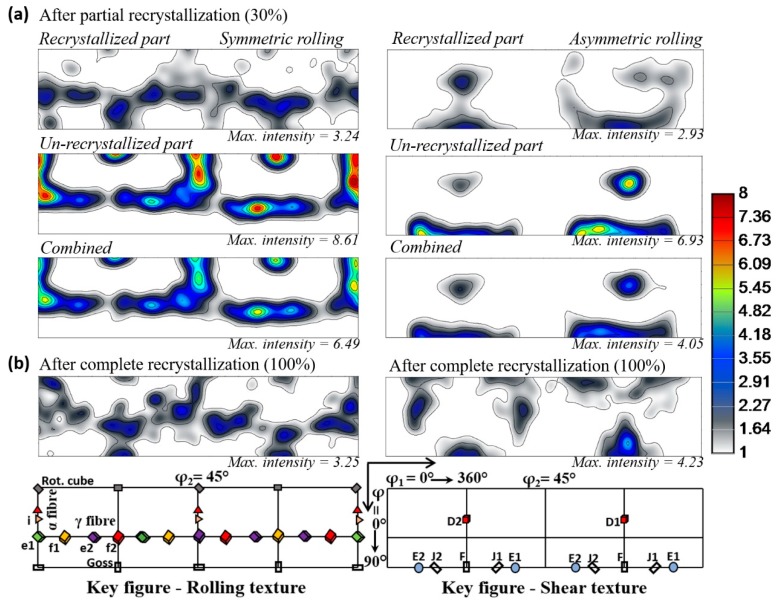
Sections of ODF for partial (**a**) and complete recrystallization (**b**) in symmetric and asymmetric rolling.

The evolution of the texture presented in Figure 17 were observed in partially (30%) and fully recrystallized specimens. For symmetric rolling, it is in accord with earlier investigations, where the prevalence of γ fiber over α fiber was reported [53,65,66,67]. However, so far in the literature, the textures of partially and fully recrystallized states have not been examined in detail for asymmetric rolling. The texture of newly formed grains in partial (30%) recrystallization consists mainly of Goss {110}<001> (or F in shear) components, while the other components are disappearing. In the fully recrystallized state, the cube or rotated cube {100}<110> components appear in proximity to its preferred ideal location along with the pre-existing Goss texture component. The retention of Goss and forming cube or rotated cube texture components in the fully recrystallized state can be comprehended by their stability. It has been observed that a strong Goss texture can be formed in the shear deformation of bcc steels while in plain strain rolling it does not appear [20,37,38]. Thus, in the recrystallization state of asymmetric rolled specimen, it can become weak but would not vanish. In symmetric rolling, the Goss component can also appear during recrystallization with low intensity, see Figure 16b. In that case, its origin lies in the shear bands [40,68]. Cube or rotated cube has been considered stable end orientation and easily forms by both deformation and annealing in bcc structured steels [62,69,70,71].

The effect of imposed asymmetry on mechanical properties was evaluated for both deformed and fully recrystallized states after 100 s rapid annealing at 660 ± 10 °C. Figure 10 shows the merits of the asymmetric rolled specimens over the symmetric case. The increased strength in deformed state can be attributed to the increased refinement in average grain size due to the severity of deformation with imposed asymmetry over symmetric rolling. After complete recrystallization, in both deformation states, there was a loss in tensile strength but a gain in ductility. Nevertheless, the loss in tensile strength after deformation is higher at imposed asymmetry than in its absence (at ratio 1:1). This is due to the extra withhold time of annealing after their complete recrystallization accomplished in 60 s, as compared to 100 s for the symmetric rolling. However, the tensile strength of asymmetric rolled specimens is still higher over the symmetric rolling, while achieving a similar level of ductility.

## 5. Conclusions

In this work, extra-low carbon steel was systematically cold rolled in single pass by varying both the applied asymmetric ratio and thickness reduction per pass. An experimental attempt was made to connect the rotation of the rolling texture to form shear texture and the measured values of the shear coefficients. The applied asymmetric conditions significantly affected both the deformed and recrystallized states. On the basis of the experimental results, we can draw the following conclusions:

1. The rolling texture approaches the shear texture when the applied thickness reduction per pass is 50% or above, together with an asymmetry ratio of 1:2.
2. The value of the shear coefficient is independent of the applied number of passes but sensitive to its measurement position along the thickness of the sheet. This measured value in a given pass with inserted new pin can be related to the expected rotation of the texture to be achieved in asymmetric rolling.
3. Under effective asymmetric conditions, there is more grain fragmentation in the mid thickness area of a sheet due to the increased lattice rotation inherent in shear compared to the symmetric case.
4. Both average grain size and JMAK exponent remain smaller in the case of asymmetric rolling than for symmetric rolling after recrystallization.
5. A very different recrystallization texture forms in optimal asymmetric rolling conditions compared to the conventional recrystallization textures observed in symmetric rolling.

## Figures and Tables

**Table 1:** Rolling schedule for different roll diameter ratios.

	Roll Diameters Ratio	1:1	1:1.3	1:1.6	1:2
Thickness Reduction per Pass					
20%		×	-	-	×
30%		×	-	-	×
40%		×	-	-	×
50%		×	×	×	×
57%		×	×	×	×
65%		×	-	-	×
75%		×	×	×	×
